# Correction to: scAnno: a deconvolution strategy-based automatic cell type annotation tool for single-cell RNA-sequencing data sets

**DOI:** 10.1093/bib/bbag462

**Published:** 2026-09-03

This is a correction to: Hongjia Liu, Huamei Li, Amit Sharma, Wenjuan Huang, Duo Pan, Yu Gu, Lu Lin, Xiao Sun, Hongde Liu, scAnno: a deconvolution strategy-based automatic cell type annotation tool for single-cell RNA-sequencing data sets, *Briefings in Bioinformatics*, Volume 24, Issue 3, May 2023, bbad179, https://doi.org/10.1093/bib/bbad179

In the originally published version of this manuscript, Formula 1 and Formula 5 contained errors.

The corrected Formula 1 should read:


(1) \begin{equation*}{\displaystyle \begin{array}{l} FC=\left\{{FC}_i|i\ \mathrm{from}\ 1\ \mathrm{to}\ m\right\}\ \mathrm{where}\\ {}{FC}_i=\frac{{\mathrm{X}}_i}{\sum_{j=1}^n{X}_{ij}-{X}_i}\cdot \left(\mathrm{n}-1\right)\end{array}} \end{equation*}

Instead of:


(1) \begin{equation*}{\displaystyle \begin{array}{l} FC=\left\{{FC}_i|i\ \mathrm{from}\ 1\ \mathrm{to}\ m\right\}\ \mathrm{where}\\ {}{FC}_i=\frac{{\mathrm{X}}_i}{\sum_{j=1}^n{X}_{ij}-{X}_i}\cdot \left(\mathrm{j}-1\right)\end{array}} \end{equation*}

The corrected Formula 5 should read:


(5) \begin{equation*} \sigma =\sqrt{\frac{1}{k}\ \sum_{i=1}^k\left({Y}_i-\overline{Y}\right)^{2}} \end{equation*}

Instead of:


(5) \begin{equation*} \sigma =\sqrt{\frac{1}{k}\ \sum_{i=1}^k\left({Y}_i-\overline{Y}\right)} \end{equation*}

These details have been corrected only in this correction notice to preserve the version of record.

